# Electrocardiographic Patterns and Arrhythmias in Cardiac Amyloidosis: From Diagnosis to Therapeutic Management—A Narrative Review

**DOI:** 10.3390/jcm13185588

**Published:** 2024-09-20

**Authors:** Lucio Teresi, Giancarlo Trimarchi, Paolo Liotta, Davide Restelli, Roberto Licordari, Gabriele Carciotto, Costa Francesco, Pasquale Crea, Giuseppe Dattilo, Antonio Micari, Michele Emdin, Antonio Berruezo, Gianluca Di Bella

**Affiliations:** 1 Department of Clinical and Experimental Medicine, University of Messina, 98100 Messina, Italy; 2 Heart Institute, Teknon Medical Centre, 08022 Barcelona, Spain; 3 Health Science Interdisciplinary Center, Scuola Superiore Sant’Anna, 56127 Pisa, Italy; 4 Departamento de Medicina UMA, Área del Corazón, Hospital Universitario Virgen de la Victoria, Centro de Investigación Biomédica en Red de Enfermedades Cardiovasculares (CIBERCV), IBIMA Plataforma BIONAND, 29010 Malaga, Spain; 5 Department of Biomedical and Dental Sciences and Morphological and Functional Imaging, University of Messina, 98100 Messina, Italy; 6 Fondazione Toscana Gabriele Monasterio, 56124 Pisa, Italy

**Keywords:** cardiac amyloidosis, arrhythmias, electrocardiogram

## Abstract

Electrophysiological aspects of cardiac amyloidosis (CA) are still poorly explored compared to other aspects of the disease. However, electrocardiogram (ECG) abnormalities play an important role in CA diagnosis and prognosis and the management of arrhythmias is a crucial part of CA treatment. Low voltages and a pseudonecrosis pattern with poor R-wave progression in precordial leads are especially common findings. These are useful for CA diagnosis and risk stratification, especially when combined with clinical or echocardiographic findings. Both ventricular and supraventricular arrhythmias are common in CA, especially in transthyretin amyloidosis (ATTR), and their prevalence is related to disease progression. Sustained and non-sustained ventricular tachycardias’ prognostic role is still debated, and, to date, there is a lack of specific indications for implantable cardiac defibrillator (ICD). On the other hand, atrial fibrillation (AF) is the most common supraventricular arrhythmia with a prevalence of up to 88% of ATTR patients. Anticoagulation should be considered irrespective of CHADsVA score. Furthermore, even if AF seems to not be an independent prognostic factor in CA, its treatment for symptom control is still crucial. Finally, conduction disturbances and bradyarrhythmias are also common, requiring pacemaker implantation in up to 40% of patients.

## 1. Introduction

Cardiac amyloidosis (CA) is an emergent cardiomyopathy related to misfolded protein deposition, most commonly transthyretin (transthyretin amyloidosis, ATTR) or light chains (light chain amyloidosis, AL). CA has an important impact on quality of life [1,2] and classically presents with an infiltrative–restrictive pattern, especially affecting diastolic function and causing heart failure with preserved ejection fraction (HFpEF). It is not limited to left ventricle dysfunction and many systemic manifestations have been described [3,4,5]. The electrocardiogram is a simple and cost-effective tool which plays an important role in suspecting CA. Furthermore, patients with CA may also frequently experience different types of arrhythmias: bradyarrhythmias caused by the involvement of the conduction system, such as atrioventricular blocks, and tachyarrhythmias, such as atrial fibrillation or ventricular arrhythmias with potentially serious outcomes. Despite their importance, ECG abnormalities and arrhythmias in CA are still poorly explored.

## 2. ECG Typical Patterns

In the 1950s, the first description of ECG patterns of CA was characteristic of the disease: low voltages in peripheral leads, poor R-wave progression in precordial leads and a pseudonecrosis aspect. QRS axis deviations, atrial fibrillation, and atrioventricular conduction abnormalities were also described [6]. ECG alterations are related to amyloid burden [7], as well as the incidence of arrhythmias [8,9,10].

**Figure 1:**
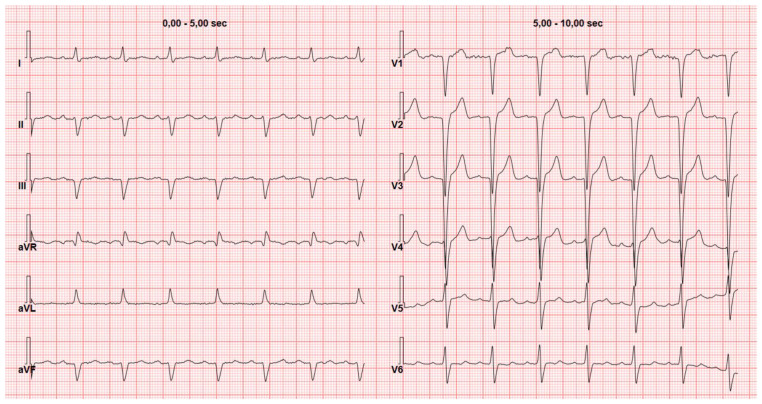
Anterior pseudonecrosis. Electrocardiogram of a 54-year-old male with variant transthyretin amyloidosis (Glu89Gln mutation) reveals left bundle branch block and anterior pseudonecrosis characterized by Q waves in leads V1–V4 measuring at least 1 mV. The patient has no history of ischemic heart disease and no kinetic abnormalities on echocardiography.

The pseudonecrosis pattern consists of the presence of Q waves measuring at least 1 mV in at least two contiguous leads without a history of ischemic heart disease and/or akinetic or dyskinetic segments in the left ventricle (LV) (Figure 1). It can be found in up to 70% of patients with CA without significant differences between transthyretin amyloidosis (ATTR) and AL [11].

**Figure 2:**
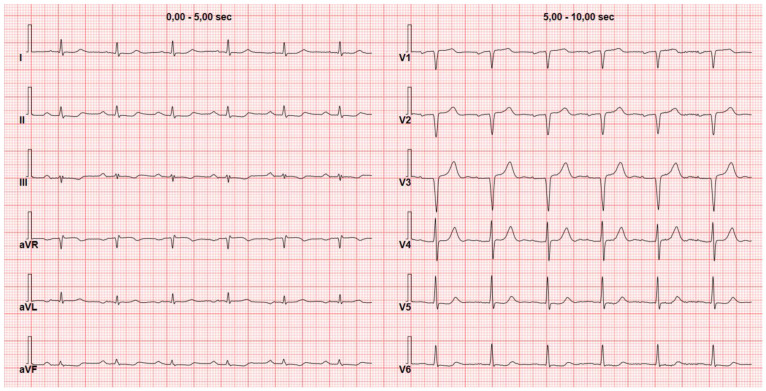
Low voltages in the peripheral leads. Electrocardiogram of a 72-year-old man with wild-type transthyretin cardiac amyloidosis and low voltages in the peripheral leads (≤5 mV in all peripheral leads) but relatively preserved voltages in the precordial leads.

A second red flag for CA on ECG is low QRS voltage (Figure 2). This phenomenon is observed in in 25–90% of CA patients with a great variability among different studies and is more frequent in AL amyloidosis compared to other types of CA [12,13,14,15]. Another potential factor contributing to the diverse prevalence of low voltages in CA is the heterogeneous definition of “low voltages”. A study has demonstrated that the prevalence of low voltages can vary within the same patient population when different criteria are used, such as a Sokolow–Lyon index of ≤15 mV, low voltages in peripheral leads (≤5 mV in each peripheral lead), or low voltages across all leads (QRS ≤5 mV in each peripheral lead and ≤10 mV in each precordial lead) [16]. Among these, the Sokolow–Lyon index is the less commonly used criteria showing low voltages in around 60% of patients with AL or ATTR in a study with 200 CA patients by Cyrille et al. [17] On the other hand, with the same criteria, a low variability of low voltage prevalence has been described ranging from 11% to 82% of patients [16,18,19,20]. It has been observed that factors influencing QRS voltages in the general population, such as left ventricular (LV) mass index, age, gender, hypertension, body surface area, and smoking habits, do not predict voltage amplitude in patients with cardiac amyloidosis (CA). This lack of correlation can be explained by the infiltration of amyloid. Indeed, these non-conductive deposits cause LV wall thickening disrupting the relationship between QRS voltages and LV mass. Consequently, the suspicion of CA should arise when there is a discrepancy between LV mass and QRS voltages [21].

Furthermore, a study found that a ratio of the total QRS score (sum of QRS amplitude in all 12 ECG leads) to left ventricular wall thickness < 92.5 mV/cm was a reliable predictor of cardiac amyloidosis in men with a bundle branch block, demonstrating 100% sensitivity and 83% specificity [22].

The combined presence of pseudonecrosis and low QRS voltages has been considered a possible diagnostic tool for CA. It has been observed that the combination of these two signs is more common in patients with systemic amyloidosis and cardiac involvement than controls (28.0% vs. 2.3%), with 28% sensitivity, 98% specificity, 96% positive predictive value and 39% negative predictive value [23]. The diagnostic performance improves then considering the amplitude of single waves and further when comparing this amplitude with other ECG parameters: a sum of S-wave amplitude in V1 plus R-wave amplitudes in V6 lower than 1.2 mV allow for the diagnosis of CA with a 91% sensitivity and an 89% specificity. The combination of ECG and echocardiographic findings has yielded the best results. Indeed, an abnormal ECG and left ventricular septal thickness > 14 mm has a 78% sensitivity and an 89% accuracy for CA diagnosis. A ratio of <0.4 between R-wave amplitude in lead I and posterior wall thickness has a 91% sensitivity and 89% specificity [24,25]. According to these results, Lofbacka et al. recently observed that a ratio of R-wave amplitude in aVR and relative wall thickness at transthoracic echocardiography > 0.9 is related to a 100% sensitivity and 95% specificity for ATTR-CA diagnosis in patients with heart failure and diastolic interventricular septum >14 mm [20]. Furthermore, Arnberg et al. found that a ratio between relative wall thickness and S-wave amplitude in aVR > 0.7 has a 97% sensitivity and 90% specificity for ATTR-CA diagnosis in patients with diastolic interventricular septum > 14 mm [26].

## 3. ECG Patterns in AL vs. ATTR Amyloidosis

CA can present with different ECG manifestations (Table 1 and Table 2).

ECG findings may assist in the differential diagnosis between AL and ATTR-CA, although they do not provide conclusive answers.

The pseudoinfarction pattern is a relatively common finding observed in about 50% of patients at diagnosis (Table 1). Cyrille et al. explored pseudoinfarction localization in inferior, lateral, and anterior leads: patients with AL had a higher incidence of anterior site localization, while ATTR patients (v and wt) had a higher incidence of inferior localization [17]. Orini et colleagues recently showed interesting differences between ATTR and AL at epicardial mapping, combining high-density superficial ECG signals and magnetic resonance sequences. At epicardial mapping, AL patients showed lower signal amplitude, greater epicardial signal fractionation, and higher dispersion of repolarization when compared to ATTR patients. Signal amplitude was negatively related to T1, whereas both signal fractionation and repolarization time increased with ECV [37].

Some studies have reported a 25% prevalence of low voltages among patients with ATTRwt versus 60% among patients with AL amyloidosis (Table 2). Therefore, the voltage-to-mass ratio is higher in patients with ATTRwt than in those with AL. Furthermore, patients with ATTRwt are also those who have a higher LV wall thickness at echocardiography. A possible explanation for these apparently conflicting results is the greater cardiomyocyte toxicity of light chains. This toxicity plays a significant role since the early phase of the disease, regardless of LV wall thickness itself. For both AL and ATTR, the low voltages prevalence is related to the severity of cardiac involvement [17]. Different findings have emerged from studies that have stratified patients with ATTRv into different groups based on the specific mutation. For example, a study has shown that 56.7% of patients with the V122I mutation have low QRS voltages. Moreover, 25% of patients met the criteria for the diagnosis of LV hypertrophy, and 56% had a first-degree atrioventricular block, compared to 20% in AL amyloidosis, as reported in other studies [18].

## 4. ECG Prognostic Role

The role of ECG findings in risk stratifications for patients with CA has been a subject of debate. In a study by Austin et al. in patients mostly diagnosed with AL amyloidosis, only the NYHA class displayed an independent association with mortality [28]. In a study by Zhao et al. among 110 patients with AL, those with pseudonecrosis waves had higher N-terminal fraction of pro-B-type natriuretic peptide (NT-proBNP) levels and were more symptomatic for dyspnea. Only Q waves and the New York Heart Association (NYHA) class emerged as independent predictors of survival [11]. On the other hand, in some studies, measures of the voltage-to-mass ratio predicted disease progression, hospitalizations for heart failure, and pacemaker implantation [12,13,14,15]. In other studies, a pseudonecrosis pattern [11], a history of myocardial infarction, and a recent syncope [30] emerged as independent predictors of cardiac death. In a recent study of 411 CA patients (AL = 120, ATTR = 291), low QRS voltages, defined as QRS ≤ 5 mV in all peripheral leads, were independently associated with cardiovascular death, both in AL and ATTR. Furthermore, when considered in combination with the National Amyloidosis Centre (NAC) staging, low QRS voltage significantly improved its prognostic value in ATTR with an area under the curve rising from 0.83 [95% CI: 0.77–0.89] to 0.87 [95% CI: 0.81–0.93] (*p* = 0.040) [13]. It has been proposed to combine ECG findings with other clinical and instrumental variables. Seven continuous variables associated with CA (systolic arterial pressure < 130 mmHg; PR duration > 200 ms; Sokolow index < 12 mV; diastolic LV posterior thickness > 13 mm; E-E’ ratio >10; global longitudinal strain > −12% and the sum of basal longitudinal strain > −47%) were selected, creating a global score with diagnostic and prognostic relevance. A cut-off of 3 showed a 90% sensitivity and 81% specificity for CA diagnosis, and the specificity became 100% with a score >5. A score >3 was related to higher mortality during follow-up (*p* < 0.001) [32]. QRS fragmentation, defined as the presence of a notched QRS complex (RsR’ pattern) without QRS prolongation [12,13,14,15], and a QRS-T angle > 102°, defined as the absolute value of the difference between the frontal plane QRS axis and T axis, were also described as prognostic ECG findings in AL-CA [38].

## 5. Atrial Arrhythmias: Atrial Fibrillation

### 5.1. Epidemiology

Atrial arrhythmias are frequent in patients with CA, and atrial fibrillation (AF) is the most common, but its prevalence is highly variable according to the etiology of CA (Table 3) [6,39]. In AL, AF has usually a lower prevalence between 15 and 20% [23,27,28,40], while in ATTR, its prevalence may be up to 88%, and it is higher in ATTRwt than in ATTRv [41,42,43]. For example, in a recent study by Cappelli et al. AF had a prevalence of 30%, 20%, and 6% respectively in ATTRwt, ATTRv and AL [14]. This discrepancy among CA subtypes may be explained by considering that patients with ATTRwt are usually older than those with ATTRv or AL. Indeed, in addition to specific abnormalities caused by the disease, which predispose to AF, aging and comorbidities such as hypertension and diabetes, may contribute themselves to the development of AF [10].

### 5.2. Pathophysiology

**Figure 3:**
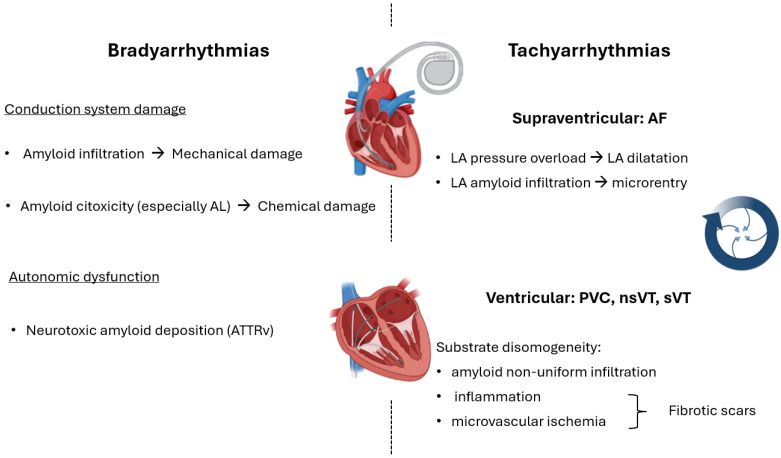
Arrhythmia pathophysiology in cardiac amyloidosis. Brady- and tachyarrhythmias are common in cardiac amyloidosis for many specific pathophysiological reasons. The main ones are summarized in the figure. AF: atrial fibrillation; AL: light chain amyloidosis; ATTRv: variant transthyretin amyloidosis; PVC: premature ventricular complex; nsVT: non-sustained ventricular tachycardia; sVT: sustained ventricular tachycardia.

In CA, many factors are involved in AF onset: atrial amyloid infiltration with electrical and mechanical impairment promoting re-entrant circuits [44,45], pressure overload caused by high left ventricular end-diastolic pressure because of ventricular diastolic dysfunction leading to left atrium dilation, ectopic beats, and electrical instability (Figure 3). As for electric abnormalities, prolongation of the AH and HV intervals is observed, along with a heterogeneous reduction of electrical voltages in the left atrium. Indeed, among 7 patients with CA (4 with ATTRwt and 3 with AL) and persistent AF undergoing an electrophysiological study with electroanatomical mapping of the left atrium for arrhythmia ablation, mean voltages per atrial segments explored (measured while on AF) were significantly lower than the same measures performed in patients with persistent AF but without CA [10]. Because of these large abnormalities, non-pulmonary vein triggers for AF are common. In a study with 74 patients undergoing AF ablation for the first time, 51 patients (68.9%) had triggers in the left atrial appendage, coronary sinus, crista terminalis, interatrial septum, or mitral valve annulus [50].

### 5.3. Prognosis

AF prognostic role in CA is still debated. It has been described as a negative prognostic factor in AL, but not in ATTR probably because in ATTR AF often occurs in patients who have already an advanced heart failure, which still remains the main prognostic factor [46,51]. For example, in a retrospective cohort study with 382 ATTR patients of whom 265 (69%) had AF, Donnellan and colleagues did not find differences in mortality between patients with AF and those without AF during a mean follow-up of 35 months (65% vs. 49%; *p* = 0.76) [52].

The monitoring of atrial arrhythmias in CA should include an ECG at each follow-up visit every 6–12 months according to the patient’s overall clinical condition. New symptoms such as recurrent palpitations or worsening fatigue should be considered red flags for potential atrial arrhythmias, especially new-onset atrial fibrillation, which should be further investigated using a 24-h Holter ECG.

### 5.4. Treatment: Rate and Rhythm Control

Data on rate and rhythm control in CA are limited, and both are more difficult to achieve in CA than in the general population. Anti-arrhythmic drugs for rate control are usually poorly tolerated. Especially, non-dihydropyridine calcium channel blockers and beta blockers use is limited by symptomatic hypotension caused by the combination of their effect with autonomic dysfunction, which is common in CA. On the other hand, digoxin use is limited by the risk of drug toxicity because in vitro amyloid fibrils bind digoxin, as confirmed by the high incidence of in vivo adverse events (12% of patients with adverse events in a 62-patient population [53]). Therefore, digoxin use should be carefully monitored and limited to patients who do not tolerate beta blockers. Finally, an ablate and pace strategy can be considered when the previous strategies are ineffective.

On the other hand, rhythm control efficacy and benefits are debated. Donnellan et al. showed that among 113 patients undergoing direct current cardioversion, 49 patients (42%) remained in sinus rhythm after one year. Patients with an early disease (stage I and II of the NAC score) demonstrated a significantly higher rate of maintaining sinus rhythm (*p* < 0.0001). Furthermore, patients who maintained sinus rhythm during the 35-month follow-up had a lower mortality compared to patients who returned in AF (43% vs. 69%, *p* = 0.003). Nonetheless, comparing patients with or without AF at baseline (117 vs. 265), no differences in mortality were observed. These findings suggest that even though AF is not a predictor of prognosis in CA, patients with early disease are the best responders to rhythm control therapy [52].

Amiodarone chronic use for rhythm control did not show mortality reduction compared to rate control [45].

To date, small studies have investigated the role of catheter ablation in CA with heterogeneous results. In 2019, Donnellan et al. studied 72 patients with ATTR randomized in a 1:2 ratio to undergo either catheter ablation or receive medical therapy. During a mean follow-up of 39 months, they observed sinus rhythm at the end of the follow-up in 9/14 patients with NAC stage I or II who underwent catheter ablation, which was against 1/10 patients with NAC stage III (*p* = 0.005). Furthermore, in this study, ablation was associated with fewer hospitalizations for heart failure or arrhythmias (*p* = 0.005) and better survival (*p* = 0.02) [47]. On the other hand, Dale and colleagues observed an 86% AF recurrence rate in 10 ATTR patients treated with catheter ablation over a 6.2-month median follow-up [54], Barrett et al. observed AF recurrence in 5/8 ATTR patients over a median follow-up of 3.2 years [55]. Recently, Maury and colleagues described eight AF recurrences at a median of 3.5 months in 22 CA patients (10 with paroxysmal AD and 12 persistent AF) [56].

In 2023, La Fazia et al. reported that 88% of 74 patients treated with pulmonary vein, left atrial posterior wall, and superior vena cava isolation were arrhythmia-free after one year [50]. On the other hand, in the same year, Ullah et al. in a population of 616 patients (293 with CA and 323 without CA) with 30-day readmission after AF catheter ablation observed higher adverse clinical events (*p* < 0.01), in-hospital mortality (*p* = 0.017), and pericardial effusion (*p* = 0.001) in patients with CA than in patients without CA [57]. These results highlight the frailty of CA patients. Poor data are available on the effects of disease-modifying therapies on arrhythmias in CA, and they are mainly focused on AF. Girvin et al. recently retrospectively observed in a population of 473 patients that tafamidis is associated with a lower incidence of AF with a hazard ratio of 0.43 (*p* = 0.003), with no difference observed among paroxysmal and persistent AF (*p* = 0.99) [58]. On the other hand, chemotherapy for AL treatment can be related to a major risk of arrhythmias and especially AF, as observed for carfilzomib [59].

Atrial flutter in CA is poorly investigated. However, as in other cardiomyopathies, catheter ablation for atrial flutter rhythm control is a feasible and effective approach [56]. In 2021, a small single-center study observed 14% of typical atrial flutter recurrence after 60.9 months of mean follow-up since the procedure [51].

### 5.5. Treatment: Anticoagulation in Atrial Fibrillation and Thromboembolism

**Figure 4:**
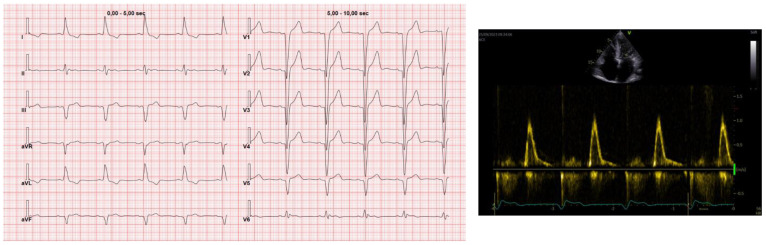
Mechanical impairment on sinus rhythm. Electrocardiogram and echocardiographic mitral pattern of an 82-year-old man with wild-type transthyretin amyloidosis (ATTR−wt). Absence of a clear A wave despite sinus rhythm being observed.

Even though anticoagulants are typically prescribed to the general population with AF based on the CHADS-VA score, specific recommendations are necessary for patients with CA because this disease is associated with a prothrombotic state, even in the presence of sinus rhythm. Therefore, the latest ESC expert consensus on CA and the most recent ESC guidelines on AF recommend anticoagulation for patients with CA and AF, regardless of the CHADS-VA score. Additionally, they advise to consider anticoagulation therapy even for patients in sinus rhythm [60,61]. Furthermore, some authors recommend transesophageal echocardiography in all patients with CA before cardioversion [62]. Vilches and colleagues studied a large population (*n* = 1191) of patients with ATTR cardiomyopathy (ATTR-CM) and observed 162 thromboembolic events during 19.9 months of follow-up, with 0 events in patients with sinus rhythm taking oral anticoagulants (OAC), 1.3 events per 100 patients-year among those with sinus rhythm without OAC, 1.7 events per 100 patients-year in AF with OAC, and 4.8 events per 100 patients year in AF without OAC [63]. Furthermore, no correlation between CHADS-VASc score and left atrial appendage thrombus has been shown in ATTR-CM [64]. The high thromboembolic risk in CA is probably caused by multiple mechanisms: left atrium remodeling and kinetic loss with blood stasis despite sinus rhythm (Figure 4), endomyocardial injury, endothelial dysfunction, local inflammation, and a hypercoagulability state both in ATTR [63], and in AL. In AL especially, the loss of anticoagulant factors because of nephrotic syndrome and inflammation caused by amyloid cardiotoxicity is also involved. To date, data do not suggest a better efficacy of OAC vs. warfarin [63], even though targeting INR could be difficult in elderly and frail patients. Left atrial appendage closure is under investigation in CA. Recently, the CAMYLAAC study demonstrated no difference in mortality between patients with and without CA undergoing this procedure, despite older age and higher comorbidities of patients with CA [65].

## 6. Ventricular Arrhythmias

### 6.1. Epidemiology

In patients with CA, ventricular arrhythmias are more common than in general population, especially in patients with advanced disease and in those with AL rather than ATTR. These arrhythmias range from isolated ventricular ectopic beats to life-threatening tachycardias.

Among 12,139 patients with amyloidosis from the Taiwan National Health Insurance Research Database, VT was 7.9 times more common than in a propensity score-matched population. Furthermore, considering only CA patients VT was 153 times more common than in the propensity score-matched population. Heart failure, diabetes, chronic liver disease, and the use of anti-arrhythmic drugs were risk factors for new-onset VT events, which in turn were associated with a worse prognosis [66]. These findings are also supported by a recent study by Patel and colleagues who observed in 168 patients with ATTR that ventricular arrhythmias (sustained or non-sustained) were associated with a more advanced NYHA class, lower ejection fraction, and a higher rate of hypertrophy at echocardiography [67].

In a study on 51 patients with AL amyloidosis, a high frequency of ventricular arrhythmias was observed on Holter ECG recording: ventricular ectopy in 29% of patients, non-sustained ventricular tachycardia (NSVT) in 18% of patients, and complex ventricular arrhythmias in 57% of patients (Lown class 3-4a-4b: 57%, class 4a-4b: 33%). Over a median follow-up of 23 months, a high mortality was observed (70%), and 25% of patients experienced a sudden cardiac death (SCD). Sustained ventricular arrhythmias were linked to a poorer prognosis and were also correlated with more significant declines in echocardiographic measures [68].

Non-sustained ventricular tachycardias (NSVT) are common both in AL and in ATTR, with a prevalence ranging from 5% to 27% in AL and around 17% in ATTR, even if specific data in ATTR are limited [69]. AL amyloidosis undergoing autologous stem cell transplantation is a well-studied subpopulation because of the high intensity of care required for these patients. In a population of 24 patients followed during their hospitalization in a stem cell transplant unit with telemetric monitoring, the patients had a high incidence of ventricular arrhythmias (326 events over 24 days on average) [70]. These events were mostly NSVT, with only one case of sustained VT, and when normalized for the length of hospital stay per patient, they were related to stroke volume and NT-proBNP levels [70].

### 6.2. Pathophysiology

Many arrhythmogenic substrates for ventricular arrhythmias can be identified: amyloid non-uniform infiltration in extracellular space, amyloid cytotoxicity, tissue inflammation, and microvascular ischemia cause electrical cardiac inhomogeneity (Figure 3). Furthermore, the same phenomena are responsible for non-uniform cardiac denervation causing a patchy adrenergic receptor upregulation, which is another substrate for ventricular arrhythmias. Due to this involvement of the heart by amyloid, the electrophysiological substrate of ventricular arrhythmias is complex and includes signal fractionation, slow intraventricular conduction, prolonged and dispersed repolarization, and anatomical and/or functional barriers able to sustain re-entrant arrhythmias. These alterations have been described as more evident in patients with AL [37]. As demonstrated by histology, ventricular arrhythmias correlate with the extent of conduction tissue infiltration [71,72]. However, the use of the extent of LGE uptake as a surrogate to predict the arrhythmogenic risk is still controversial, probably because it is not simple to distinguish between fibrosis and amyloid using LGE sequences in CMR. On the other hand, the use of other CMR sequences for non-invasive quantification of amyloid burden and thus for arrhythmogenic evaluation is promising [67]. Finally, CA is a major cause of heart failure, which can promote ventricular arrhythmias via different nonspecific mechanisms: structural remodeling related to increased workload and ventricular pressure; electrical remodeling because of an abnormal ion channel expression, especially for potassium currents; and neurohormonal activation, including elevated levels of catecholamines and angiotensin II, which enhance myocardial excitability, thereby promoting electrical instability.

### 6.3. Prognosis and Treatment

According to different studies, up to one-half of CA patients die suddenly. The underlying cause for the high rate of SCD in patients with cardiac amyloidosis (CA), as well as the specific types of arrhythmias responsible for this outcome, remain unclear [8].

The prognostic role of NSVT is still debated, and unfortunately, there is a lack of data on NSVT management in CA. A recent metanalysis by Halawa et al. with 194 patients with CA and an implantable cardiac defibrillator (ICD) (AL = 108, ATTR = 70, serum amyloid A amyloidosis = 16) showed a 51% prevalence of NSVT and appropriate ICD shock in 18% of patients, but the correlation between these events remains to be investigated [73].

The electrophysiological study probably has a role in SCD risk stratification in CA patients. In a population of 25 AL patients, Reisinger and colleagues showed that CA is often associated with a long infra-his (HV > 55 ms) conduction time (23–25 patients), which was a predictor of SCD (*p* = 0.05), while VT inducibility was not correlated to SCD (*p* = 0.77) [74]. The same group also demonstrated that in a population of 133 patients with AL amyloidosis during a median 44-month follow-up (range of 24 to 91 months), abnormal signal-averaged ECG (SAECG, QRS ≥ 115 ms, root mean square voltage < 25 µV, low amplitude signals duration > 38 ms) was correlated to cardiovascular death (*n* = 71), especially to SCD (*n* = 33, *p* < 0.0001) [75]. This has a rationale in the fact that SAECG gives information on ventricular activation heterogeneity and on the presence of delayed potential, which are correlated to both advanced disease and ventricular arrhythmias. However, in this study, 53% of patients with SCD did not show abnormal SAECG, suggesting that in CA, not only ventricular arrhythmias but also other mechanisms such as electro-mechanical dissociation or bradyarrhythmias are involved in SCD.

According to the latest ESC guidelines, premature ventricular complex treatment (PVC) is recommended when PVCs are symptomatic (class I) and should be considered when PVCs have a burden greater than 10% and are responsible for tachycardiomyopathy or when they limit biventricular pacing in nonresponders to cardiac resynchronization therapy (CRT) (class IIa). It could be treated even in asymptomatic patients with a burden greater than 20% (class IIb). Catheter ablation or medical treatment with beta blockers or non-dihydropyridine calcium channel blockers is highly dependent on the expertise of the individual center, with invasive treatment especially recommended for PVC with an origin from the right ventricular outflow tract [76]. A wise approach for NSVT screening and follow-up should be performed, and an annual Holter ECG could be considered to investigate PVC burden in patients without an implantable device. Furthermore, PVCs clinical impact should be investigated at each follow-up visit.

Conversely, studies based on the interrogation of defibrillators or loop recorders showed that the causes of SCD in CA patients are predominantly electromechanical dissociation or bradyarrhythmias [40,77,78]. Therefore, risk factors for malignant ventricular arrhythmias in CA remain to be elucidated [79] and specific ICD indications are still general: according to the last Heart Rhythm Society expert consensus (HRS) on this field, ICD implantation is recommended in patients with CA who survived to cardiac arrest (class I) and can be considered in primary prevention in AL patients with NSVT (class IIB). In both cases, life expectancy should be greater than one year [80].

## 7. Bradyarrhythmias and Conduction Disturbances

### 7.1. Epidemiology

Conduction abnormalities are some of the most commonly reported findings in patients with CA that particularly involve the atrioventricular node and the ventricular myocardium, despite a relative preservation of the sinus node and specialized conduction tissue (Table 4). Recently, in a cohort study with 261 ATTRwt patients and 108 ATTRv patients, Donnellan et al. reported a sick nodus disease prevalence of 7%, with no significant differences between ATTRwt and ATTRv [41]. In a study of 25 patients with AL, sinus node function was normal in 88% of cases. Furthermore, another study showed that in 20 patients with an implantable loop recorder implanted for syncope or presyncope, only one patient experienced sinus pauses requiring cardiac pacing during the follow-up [81].

Conduction defects in the His–Purkinje system are common both in AL and ATTR, while symptomatic atrioventricular blocks are more common in ATTR patients, probably because they are older and have longer survival than AL patients. This results in a higher prevalence of device implantation in ATTR than in AL even before diagnosis of CA [14].

Comparing ATTR and AL, a small study of 14 patients showed longer HV intervals in AL than in ATTR patients with no significative differences in AH interval and QRS duration [43]. Among intraventricular conduction disturbances, right bundle branch block (RBBB) and left anterior fascicular hemiblock (LAFB) have a similar prevalence between AL and ATTR, ranging from 8 to 20% for RBBB [13,18,19,22,29,41] and from 18 to 52% for LAFB according to the study population [13,20,29,82]. A left bundle branch block (LBBB) was found in 40% of patients with ATTRwt, while it is only an occasional finding in other forms of CA [29] probably as a consequence of a greater conduction disease because of old age and related comorbidities [29,41].

In a cohort of 127 patients with AL amyloidosis confirmed via endomyocardial biopsy, 76% exhibited normal atrioventricular conduction, while 24% experienced some form of atrioventricular block, with first-degree block being the most prevalent (21%). A right bundle branch block (RBBB) was observed in 9% of the patients (Figure 3), and 5% showed a left bundle branch block (LBBB). These findings appeared to be unaffected by the extent of myocardial infiltration, which was indirectly estimated based on the degree of wall thickness [27].

QT prolongation is a common finding in CA with a prevalence of around 52% and 63% of patients, without significative differences between AL, ATTRwt, and ATTRv [17,33].

On the other hand, bradyarrhythmia in CA can be related also to autonomic dysfunction which is common and is a useful tool for differential diagnosis with other cardiac hypertrophic phenocopies. In amyloidosis, especially in AL and ATTRv, autonomic dysfunction is due to a combination of both heart failure and autonomic nervous system damage for direct amyloid deposition in peripheral nerves [83].

### 7.2. Pathophysiology

ECG abnormalities are often attributed to the direct infiltration of conduction pathways by amyloid fibrils, but findings from histological examinations do not completely agree with this hypothesis. In a series of 23 autopsies on CA patients (18 diagnosed with “senile” CA, 3 with CA related to a plasma cell dyscrasia, 1 systemic form, and another affecting primarily the peripheral nervous system) 7 cases with limited cardiac infiltration, 5 with moderate infiltration, and 11 with severe infiltration were observed. In this population a high prevalence of rhythm disturbances was noted irrespective of amyloid burden: atrial arrhythmias in 39% of patients, atrioventricular blocks in 43%, bundle branch blocks in 30%, low voltages in 35%, and poor R-wave progression in precordial leads in 30% [39]. On the other hand, cardiac fibrosis was observed in 30% of patients, with a tendency to greater severity in patients with moderate to severe amyloid infiltration. The interpretation of these findings suggests that ECG abnormalities could derive from blocks between the nodes or at the interface between conduction and working tissue irrespective of amyloid burden and that the damage to conduction tissue is at least partially reactive or toxic. Nonetheless, these conclusions are speculative, also because of the absence of more extensive histopathological analyses in these series (Figure 3).

### 7.3. Treatment

In ATTR, conduction disturbances require pacemaker implantation in 25–45% of cases [81]. In the population studied by Donnelan et al. (ATTRwt = 261, ATTRv = 108), the most common conduction disorder was a wide QRS complex (in 51% and 48% of cases respectively) with higher values observed in ATTRwt than in ATTRv (*p* = 0.02). No other significative conduction disturbance differences were observed between these two groups, neither were they observed between Val122Ile (*n* = 75) and Thr60Ala (*n* = 12) patients. First-degree atrioventricular block was also very common (49% in ATTRwt and 43% in ATTRv). Furthermore, during a median follow-up of 28 months, 11% of patients developed advanced atrioventricular block requiring pacemaker implantation without significative differences between ATTRv and ATTRwt (*p* = 0.64). Wide QRS complex at baseline was associated with the development of a high degree atrioventricular blocks at follow-up, resulting in a good marker of early infiltration of the conduction system by amyloid deposits. On the other hand, a high degree of atrioventricular block was not associated with increased mortality during the follow-up, probably acting as an epiphenomenon of the global heart failure progression [41].

In CA, conduction disturbance monitoring should include an accurate ECG analysis at each follow-up visit every 6–12 months. The most relevant implication of this follow-up is considering CRT when the need for device implantation arises in a patient with documented progressive QRS widening.

### 7.4. Prognosis

Prognostic relevance of bradyarrhythmias and conduction disturbances in CA is still uncertain due to conflicting findings from available data. In one study involving 59 patients with CA (43 with AL and 16 with ATTRv) with a mean follow-up of 880 ± 110 days, a shorter PQ interval and longer QRS duration were linked to a poorer prognosis. The PQ interval measured 166 ± 4.8 ms in survivors (*n* = 41) and 164 ± 5.6 ms in patients who died during follow-up (*n* = 18, *p* < 0.01), while the QRS interval was 95.7 ± 3.4 ms and 115.1 ± 6.7 ms, respectively (*p* < 0.05). There were no differences between patients with AL and those with ATTRv. A considerable number of patients exhibited right ventricular conduction disturbances (32%) or a left anterior hemiblock (20%), whereas left bundle branch block (LBBB) was rare, occurring in only one patient [84]. These results are consistent with those observed by Boldrini et al. in a population of 344 patients with AL, of which 240 with cardiac involvement and 104 without. Patients with AL-CA had longer PQ (179 ± 37 vs. 169 ± 30 ms, *p* = 0.023) and QRS intervals (90 ± 21 vs. 85 ± 20 ms, *p* = 0.022), as well as a prolonged QTc interval (456 ± 37 vs. 425 ± 30, *p* < 0.001). Furthermore, there was also a trend, though not significant, toward having more atrioventricular disturbances in patients with AL-CA than in patients with AL without cardiac involvement (18% vs. 13%). Fascicular conduction disturbances were present in 28% of AL-CA patients, mostly right bundle branch block (RBBB), compared with 17% in those with isolated AL amyloidosis. Notably, the study indicated that fascicular conduction disturbances were associated with poorer survival, independent of other risk markers like NT-proBNP or troponin levels [82]. Even if these findings do not establish causality, they suggest a simple parameter to be investigated for risk stratification and could help explain why a significant proportion of deaths in these patients are due to pulseless electrical activity or electromechanical dissociation [40,78].

In a separate investigation, Reisinger et al. assessed 25 patients with AL-CA, finding that 52% of patients had a first-degree atrioventricular block, 20% had QRS prolongation, with left and right bundle branch block (LBBB and RBBB) equally prevalent at 8% each, and the anterior fascicular block was observed in 24% of patients [74]. Both atrium-His (AH) and His–ventricle (HV) intervals were prolonged (121 ± 33 ms and 79 ± 18 ms, respectively). Furthermore, all patients with a large QRS (>120 ms) had also an infrahissian delay, and five patients with a significant HV interval prolongation (>80 ms) also showed highly fragmented potential. Prolonged HV interval was also independently associated with an increased risk of sudden cardiac death (SCD) during follow-up, with an odds ratio of 2.26 for each 10 ms prolongation. A prolongation of the HV interval with preserved QRS duration has been described in CA as a consequence of amyloid infiltration of the right and left bundle branches and distal conduction systems [81].

## 8. Article Methodology and Limitations

The current article is a narrative review with epidemiology data on ECG patterns and arrhythmias in CA collected via the careful and meticulous research of studies published in PubMed until May 2024. However, other databases have not been systematically consulted, so the presence of additional articles reporting epidemiological data on the subject cannot be ruled out. Case reports and small case series (<20 patients) were excluded.

## 9. Conclusions

ECG can provide important red flags for CA diagnosis. A pseudonecrosis pattern and low QRS voltages compared to heart mass are the most suggestive ECG findings, but also other nonspecific ECG abnormalities, such as atrioventricular blocks and bundle branch blocks or arrhythmias as atrial fibrillation, PVCs, sustained and non-sustained ventricular tachycardias are common and can provide insights into the extent of cardiac involvement.

Even if still investigated in small studies, tachy- and bradyarrhythmias are common findings in CA. Their impact on prognosis is still debated; nonetheless, their treatment is a crucial part of the disease management for symptom control and to avoid complications, such as thromboembolic events in AF or traumatic syncopes for conduction disorders. AF catheter ablation efficacy compared to the general population and ventricular arrhythmias management for the prevention of SCD are important topics under investigation.

## Figures and Tables

**Table 1:** Pseudonecrosis patterns (any location) in patients with cardiac amyloidosis.

	N		Pseudonecrosis Pattern		
		AL	ATTRwt	ATTRv	AL + ATTR
Murtagh B, et al. (2005) [27]	127	-	-	-	47
Austin BA et al. (2009) [28]	45	-	-	-	
Rapezzi C et al. (2009) [29]	233	69	66	69	
Dungu J et al. (2012) [18]	64	-	-	52	
Cheng Z et al. (2013) [23]	276	-	-	-	
Finocchiaro G et al. (2013) [30]	48	-	-	-	
Mussinelli R et al. (2013) [16]	233	-	-	-	
Perlini S et al. (2013) [31]	375	-	-	-	
Rapezzi C et al. (2013) [15]	186	-	33	55	
Cyrille NB et al. (2014) [17]	200	25	18	18	
Di Bella G et al. (2015) [11]	35	-	-	28	
Zhao L et al. (2016) [11]	110	-	-	-	
Cariou E et al. (2017) [32]	114	-	-	-	68
Gonzalez-Lopez E et al. (2017) [19]	149	-	63	-	
Cappelli F et a. (2020) [14]	244	39	23	34	
Löfbacka V et al. (2021) [20]	33	-	29		
Guo X et al. (2021) [33]	99	62	-	-	
Cipriani A et al. (2022) [13]	411	-	-	-	
Pericet-Rodriguez C et al. (2022) [34]	58	-	-	-	60

N = number of patients in the study. Other data are reported as percentages. AL, light chain amyloidosis; ATTR, transthyretin amyloidosis (ATTRv, variant form; ATTRwt, wild-type form).

**Table 2:** Low-voltage pattern in patients with cardiac amyloidosis.

		Low Voltages							
	N	Peripheral				Precordial			
		AL	ATTRwt	ATTRv	AL + ATTR	AL	ATTRwt	ATTRv	AL + ATTR
Murtagh B, et al. (2005) [27]	127	-	-	-	45	-	-	-	4
Austin BA et al. (2009) [28]	45	-	-	-		-	-	-	42
Rapezzi C et al. (2009) [29]	233	60	40	25		-	-	-	
Dungu J et al. (2012) [18]	64	-	-	25		-	-	49	
Cheng Z et al. (2013) [23]	276	-	-	-	54	-	-	-	
Finocchiaro G et al. (2013) [30]	48	-	-	-	53	-	-	-	
Mussinelli R et al. (2013) [16]	233	66	-	-		38	-	-	
Perlini S et al. (2013) [31]	375	64	-	-		-	-	-	
Rapezzi C et al. (2013) [15]	186	-	-	-		-	-	-	
Cyrille NB et al. (2014) [17]	200	37	18	38		16	6	10	
Di Bella G et al. (2015) [11]	35	-	-	53		-	-	-	
Zhao L et al. (2016) [35]	110	54	-	-		54	-	-	
Cariou E et al. (2017) [32]	114	-	-	-	40	-	-	-	10
Gonzalez-Lopez E et al. (2017) [19]	149	-	22	-		-	22	-	
Cappelli F et al. (2020) [14]	244	49	27	31		-	-	-	
Li H et al. (2020) [36]	69	13	-	-		-	-	-	
Guo X et al. (2021) [33]	99	68	-			35	-	-	-
Cipriani A et al. (2022) [13]	411	55	35			-	-	-	-
Pericet-Rodriguez C et al. (2022) [34]	58	-	-		35	-	-	-	-

N = number of patients in the study. Other data are reported as percentages. AL, light chain amyloidosis; ATTR, transthyretin amyloidosis (ATTRv, variant form; ATTRwt, wild-type form).

**Table 3:** Atrial fibrillation in patients with cardiac amyloidosis.

	N	Atrial Fibrillation			
		AL	ATTRwt	ATTRv	AL + ATTR
Murtagh B et al. (2005) [27]	127	-	-	-	10
Austin BA et al. (2009) [28]	45	-	-	-	18
Dungu J et al. (2012) [18]	64	-	-	-	69
Cheng Z et al. (2013) [23]	276	-	-	-	16
Finocchiaro G et al. (2013) [30]	48	-	-	-	25
Rapezzi C et al. (2013) [44]	186	-	37	29	
Cyrille NB et al. (2014) [17]	200	6	38	17	
Longhi S et al. (2015) [28]	262	9	38	11	
Sperry BW et al. (2016) [21]	389	32	75	24	
Zhao L et al. (2016) [11]	110	12	-	-	
Cariou E et al. (2017) [32]	114	-	-	-	28
Gonzalez-Lopez E et al. (2017) [19]	149	-	56	-	
Mints YY et al. (2018) [45]	146	-	70	-	
Sidana S et al. (2019) [46]	239	14	-	-	
Cappelli F et al. (2020) [14]	244	6	40	20	
Donnellan E et al. (2020) [47]	369	-	33	13	
Löfbacka V. et al. (2021) [20]	33	-	-	33	
Guo X et al. (2021) [33]	99	24	-	-	
Cipriani A et al. (2022) [13]	411	14	-	32	
Pericet-Rodriguez C et al. (2022) [34]	58	36	-	-	
Russo D et al. (2023) [48]	64	-	-	23	
Fumagalli C et al. (2023) [49]	266	-	78	-	

N = number of patients in the study. Other data are reported as percentages. AL, light chain amyloidosis; ATTR, transthyretin amyloidosis; ATTRv, variant transthyretin amyloidosis; ATTRwt, wild-type transthyretin amyloidosis.

**Table 4:** Conduction disturbances in the different forms of amyloidosis.

	N		I Degree AV Block			II Degree AV Block			
		AL	ATTRv	ATTRwt	AL + ATTR	AL	ATTRv	ATTRwt	AL + ATTR
Murtagh B, et al. (2005) [27]	127	-	-	-	21	-	-	-	1
Rapezzi C et al. (2009) [29]	233	18	25	33		-	-	-	
Dungu J et al. (2012) [18]	64	-	56	-		-	-	-	
Boldrini M et al. (2013) [82]	344	15	-	-		-	-	-	
Cheng Z et al. (2013) [23]	276	-	-	-	13	-	-	-	
Finocchiaro G et al. (2013) [30]	48	-	-	-	21	-	-	-	
Perlini S et al. (2013) [31]	375	25	-	-		-	-	-	
Rapezzi C et al. (2013) [15]	186	-	13	30		-	-	-	
Cyrille NB et al. (2014) [17]	200	26	33	45		0	10	10	
Di Bella G et al. (2015) [24]	35	-	-	-		-	-	-	
Cariou E et al. (2017) [32]	114	-	-	-		-	-	-	
Gonzalez-Lopez E et al. (2017) [19]	149	-	-	31		-	-	-	
Cappelli F et al. (2020) [14]	244	13	41	26		-	-	-	
Donnellan E et al. (2020) [47]	369	-	43	49		-	7	10	
Löfbacka V et al. (2021) [20]	33	-	24			-	-	-	
Guo X et al. (2021) [33]	99	-	-	-		-	-	-	
Sharma S et al. (2021) [22]	71	-	-	-		-	-	-	
Cipriani A et al. (2022) [13]	411	22	48			-	-	-	
Pericet-Rodriguez C et al. (2022) [34]	58	-	-	-	32	-	-	-	

N = number of patients in the study. Other data are reported as percentages. AL, light chain amyloidosis; ATTR, transthyretin amyloidosis; ATTRv, variant transthyretin amyloidosis; ATTRwt, wild-type transthyretin amyloidosis.

## Data Availability

Data sharing is not applicable.
